# Plasmon-Induced Electrocatalysis with Multi-Component Nanostructures

**DOI:** 10.3390/ma12010043

**Published:** 2018-12-24

**Authors:** Palaniappan Subramanian, Dalila Meziane, Robert Wojcieszak, Franck Dumeignil, Rabah Boukherroub, Sabine Szunerits

**Affiliations:** 1 Department of Material Engineering, KU Leuven, Kasteelpark Arenberg 44, P.O. Box 2450, B-3001 Heverlee, Belgium; palan.subramanian@kuleuven.be; 2 Département de Chimie, Faculté des Sciences, Université Mouloud Mammeri, B.P N 17 RP, Tizi Ouzou 15000, Algérie; d_meziane@yahoo.fr; 3 Univ. Lille, CNRS, Centrale Lille, ENSCL, Univ. Artois, UMR 8181-UCCS-Unité de Catalyse et Chimie du Solide, F-59000 Lille, France; robert.wojcieszak@univ-lille.fr (R.W.); franck.dumeignil@univ-lille.fr (F.D.); 4 Univ. Lille, CNRS, Centrale Lille, ISEN, Univ. Valenciennes, UMR 8520-IEMN, F-59000 Lille, France; rabah.boukherroub@univ-lille.fr

**Keywords:** plasmonics, catalysis, nanomaterials, electrochemistry, fuel, fuel cells

## Abstract

Noble metal nanostructures are exceptional light absorbing systems, in which electron–hole pairs can be formed and used as “hot” charge carriers for catalytic applications. The main goal of the emerging field of plasmon-induced catalysis is to design a novel way of finely tuning the activity and selectivity of heterogeneous catalysts. The designed strategies for the preparation of plasmonic nanomaterials for catalytic systems are highly crucial to achieve improvement in the performance of targeted catalytic reactions and processes. While there is a growing number of composite materials for photochemical processes-mediated by hot charge carriers, the reports on plasmon-enhanced electrochemical catalysis and their investigated reactions are still scarce. This review provides a brief overview of the current understanding of the charge flow within plasmon-enhanced electrochemically active nanostructures and their synthetic methods. It is intended to shed light on the recent progress achieved in the synthesis of multi-component nanostructures, in particular for the plasmon-mediated electrocatalysis of major fuel-forming and fuel cell reactions.

## 1. Introduction

Plasmon-accelerated chemical transformation through localized surface plasmon resonance (LSPR) excitation was first experimentally observed during the photocatalytic degradation of formaldehyde (HCHO) and methanol (CH_3_OH) on gold nanoparticles (Au NPs) 10 years ago [1]. It was shown that, when irradiated with visible light, Au NPs dispersed on different metal oxide supports (ZrO_2_, Fe_2_O_3_, CeO_2_, SiO_2_) exhibit significant activity in the oxidation process. Since then, metal-based nanocomposites are widely considered for the construction of photo-redox catalysts. The ability of CdS–Pt nanostructures as photocatalysts for light-driven H_2_ production was demonstrated some years later by Wu et al. [2]. Using ultrafast transient absorption spectroscopy, it was demonstrated that the excitons in CdS dissociate by ultrafast electron transfer (∼3.4 ps) to Pt and the charge separated state is long-lived (∼1.2 ± 0.6 μs) due to hole trapping in CdS. Recently, photocatalytic water splitting using Au NPs was demonstrated using a plasmonic photoelectrode [3], where electron transfer from Au NPs to protons in water generated the photocurrent. As no semiconductor was involved in the catalytic system, no Schottky barrier was formed, and a higher collection efficiency of hot carriers was achieved. These selected examples revealed that harvesting energy from the hot charge carriers of plasmonic structures is promising for energy conversion and photocatalysis alike, paving the way for developments of various catalytic reactions. Most attention has been devoted to plasmon-mediated chemical transformations, and a few reports have investigated plasmon-enhanced electrochemical transformations [4,5,6,7,8,9,10,11,12,13,14]. The progress that has been achieved in the synthesis of materials that are useful for the direct plasmon-accelerated electrocatalysis as well as on metal/semiconductor composites will be outlined here, with the belief that the application of new concepts of electrocatalysis is essential for the advancements of many industrial electrocatalytic processes. These applications might also be of interest for fuel cells, electrochemical sensors, organic electrosynthesis, and so forth. Before describing the synthetic aspects in more detail, we briefly discuss the localized surface plasmon resonance effect and the mechanism of plasmon-enhanced electrocatalysis.

## 2. Mechanism of Plasmon-Enhanced Electrocatalysis

**Figure 1:**
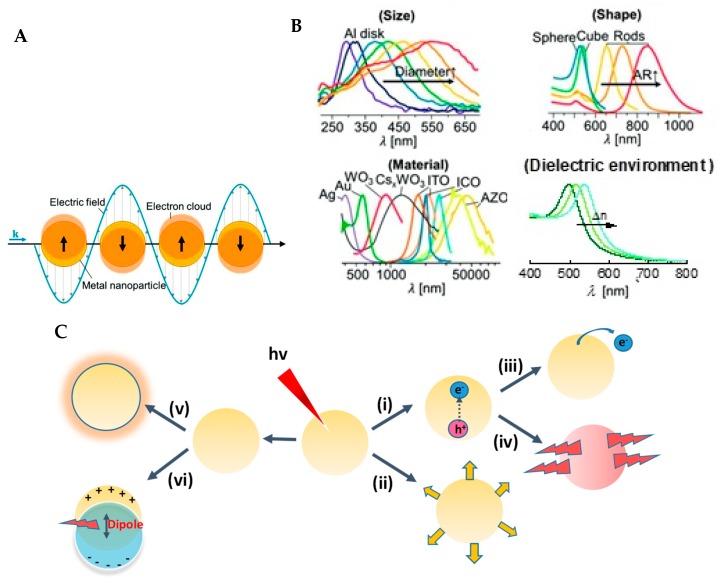
(**A**) Coherent collective oscillations of free electrons of metal nanoparticles in response to light when the diameter of the nanoparticles is smaller than the wavelength of light. (**B**) Plasmonic resonances are engineered by the size [23], shape [24], material composition of nanomaterials [25], and dielectric environment (n = 1.00 to 1.50). (**C**) The decay processes of excited surface plasmon resonance waves: (**i**) non-radiative decay by the excitations of charge carries; (**ii**) radiative decay via scattering, (**iii**) transfer of hot charge carriers to the surrounding; (**iv**) relaxation via heat transfer, (**v**) electromagnetic field enhancement, and (**iv**) dipole resonance energy transfer.

Metallic nanoparticles that have diameters smaller than the wavelength of light are known to support coherent collective oscillations of delocalized electrons in response to electromagnetic radiation, which are known as localized surface plasmons (LSPs) (Figure 1A). Upon resonant excitation, the collective oscillations of the free electrons give rise to a local electric field enhancement near the surface of the nanoparticles, which strongly concentrates light intensities. The resonances where they occur are named ‘localized surface plasmon resonances’ (LSPR) to differentiate them from the propagating surface plasmon polaritons of metal surfaces. A tremendous amount of efforts has been devoted to the design of plasmonic structures to adjust the LSPR frequency (*λ_max_*), and it is now possible to engineer nanostructures that show LSPR effects from the ultraviolet to the mid-infrared spectral zones (Figure 1B). This is possible as the LSPR properties of plasmonic nanomaterials are strongly dependent on their morphology, size, shape, composition, and even spacing between particle assemblies, allowing the use of several parameters for tuning their *λ_max_*. Since the LSPR frequency shifts upon changes of the refractive index of the medium surrounding the particles, embedding metallic nanostructures into other dielectric layers, or forming core–shell plasmonic structures are other means of tuning the LSPR effect.

Following light absorption by nanoparticles and LSPR excitation in the nanoparticles, the plasmons can decay in several competitive pathways (Figure 1C). One is the radiative decay, upon which the plasmon decays into photons, resulting in strong light-scattering effects. This phenomenon is often used for imaging applications and sensing [15,16], and is at the heart of surface-enhanced Raman spectroscopy (SERS) [17,18]. The other route is the non-radiative decay process, which is dominant for small metallic nanoparticles (<40 nm) and leads to the generation of energetic electrons and holes in the plasmonic nanostructures. The excited surface plasmons can then decay by relaxation to generate localized heating effects, which are detrimental for thermal-based applications such as photothermal therapy [19], as well as for plasmon-induced photocatalytic chemical reductions [20]. In addition, excited surface plasmons can transfer “hot” charge carriers to their surroundings, which is primordial for light-driven chemical transformations [20]. Indeed, hot-electron injection is the first mechanism reported for the plasmon-enhanced photoactivity of wide-band gap semiconductors [21,22].

Two situations have to be distinguished in the case of plasmon-mediated electrochemistry: *(1)* pure plasmonic metal nanostructures, and *(2)* metal/semiconductor composites with underlying fundamentally different mechanisms.

### 2.1. Indirect Mechanism Using Pure Plasmonic Nanostructures

**Figure 2:**
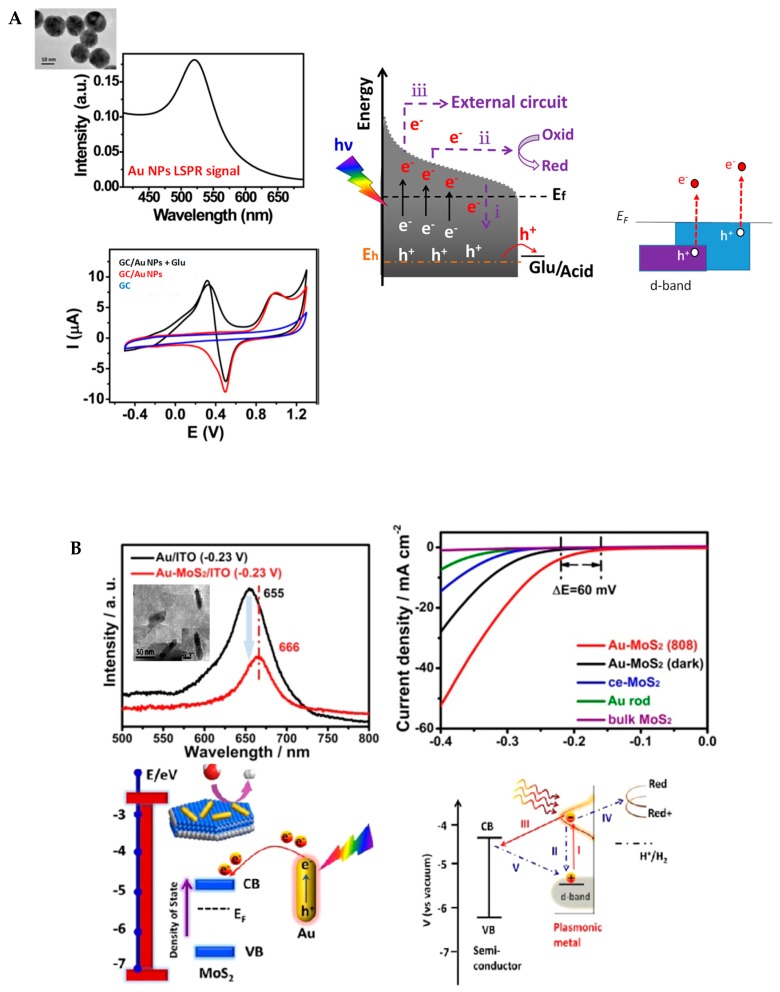
(**A**) (**a**) Localized surface plasmon resonance (LSPR) signal of gold nanoparticles (Au NPs) (inset TEM image) and cyclic voltammograms of glassy carbon (GC)/Au NPs in phosphate-buffered saline (PBS) (red) and in glucose (100 mM; black) as well as of GC in PBS (dark blue) together with mechanisms of direct plasmon-accelerated electrochemical reactions using the oxidation of glucose to gluconic acid as an example (reprinted with permission from Ref [4]); (**B**) LSPR spectra of Au rods in Au/Indium Tin Oxide(ITO) and Au−MoS_2_/ITO (inset: TEM image of Au–MoS_2_ hybrids and Au NR); polarization curves recorded on Au, MoS_2_, and Au–MoS_2_ hybrid (under illumination and dark) and energy level diagram (reprinted with permission from Ref [26]).

The plasmon-accelerated electrochemical oxidation of glucose into gluconic was reported by Wang et al. [4] (Figure 2A). An enhanced electrochemical response of glucose oxidation was observed upon the LSPR excitation of Au NPs. Taking into account light intensity, heat effect, and the influence of the LSPR wavelength, the following reaction mechanism was proposed. Upon light absorption and LSPR excitation, the electrons of the Au NPs oscillate collectively and interband excitation occurs, which results in electrons at active states above the Fermi level energy of Au NPs. This excited charge is concentrated on the surface of the Au NPs, and has three possible transfer channels: *(i)* recombination with formed holes, *(ii)* electron transfer, and *(iii)* being removed to the external circuit. In this case, the positive applied potential drives the hot electrons to the external circuit, and the remaining hot holes are driven to the Au NPs surface to accelerate glucose oxidation. This corresponds to the generation and injection of hot charge carriers into adsorbed molecules, which is often identified as an ‘indirect’ mechanism in the literature.

### 2.2. Direct Mechanism by Promotion of an Electron from the Metal to an Empty Molecular Orbital on the Adsorbate

The case of using a non-pure plasmonic substrate but a metal/semiconductor composite is outlined in Figure 2B. The hot electrons are injected from the metal nanoparticles to the conduction band of the semiconductor upon overcoming the Schottky barrier. This process enables the entrapment of hot electrons in the semiconductor particles, and thereby suppresses the electron–hole recombination, promoting redox reactions occurring on the semiconductor nanoparticles. Yi et al. recently demonstrated that the plasmon-excited hot electrons that are generated on Au NRs can be injected to a MoS_2_ layer due to the low Schottky barrier between Au NRs and MoS_2_ [26]. This system exhibited enhanced electrocatalytic activity toward the hydrogen evolution reaction (HER) due to the increase in charge density on MoS_2_ upon the injection of hot electrons. Three probable transfer pathways are described, namely: *(i)* the recombination with holes in Au NRs, *(ii)* injection into the conduction band of the MoS_2_, and *(iii)* the direct electrochemical reduction of water on Au NRs by the generated hot electrons (Figure 2B). Semiconductors are used as charge transfer mediators to efficiently collect the excited carriers and thereby promote electrochemical reactions.

## 3. Synthesis of Plasmonic Electrocatalysts: From Single to Multi-Component Nanostructures

Over the last two decades, a great deal of research has been devoted to the development of nanostructured materials for improved electrocatalysis (Table 1). It is now well established that next to morphology, the size and shape of the nanomaterials are considerably affecting the overall electrocatalytic activity of such systems [5,27]. All of the developed systems focused on electrocatalysis in the dark or under daylight, and a wealth of information on the structure/activity relationship of electrocatalysis on light-induced resonant phenomena have been neglected. Inspired by important advances in using light to enhance photocatalysis [22] as well as photoelectrocatalysis, as recently shown by Thomas et al. using a pure Au NPs plasmonic photo electrode architecture [3], similar effects are expected to be beneficial for plasmon-mediated electrocatalysis.

### 3.1. Noble Metals-Based Plasmonic Electrocatalysts

Gold (Au), silver (Ag), and copper (Cu)-based nanostructures are the most widely investigated plasmonic nanostructures for electrocatalysis, as they easily allow the tuning of the plasmon resonance from the ultraviolet-visible to the near infrared region, which are the major components of the solar flux. In addition, the advantage of such systems is that the Schottky junction commonly blocking the collection of hot carriers is avoided.

Gold nanoparticles decorating glassy carbon electrodes [4,5] as well as gold nanofiber-based electrodes [11] have been proposed for plasmon-induced electrochemical processes. Wang et al. underlined the importance of hot spots in Au NSs on direct plasmon-enhanced electrochemistry using the oxidation of ascorbic acid as a model [5]. He suggested that an increased number of hot spots, as observed on gold nanostars (Au NSs), results in the best plasmon-enhanced electrochemistry effects.

**Figure 3:**
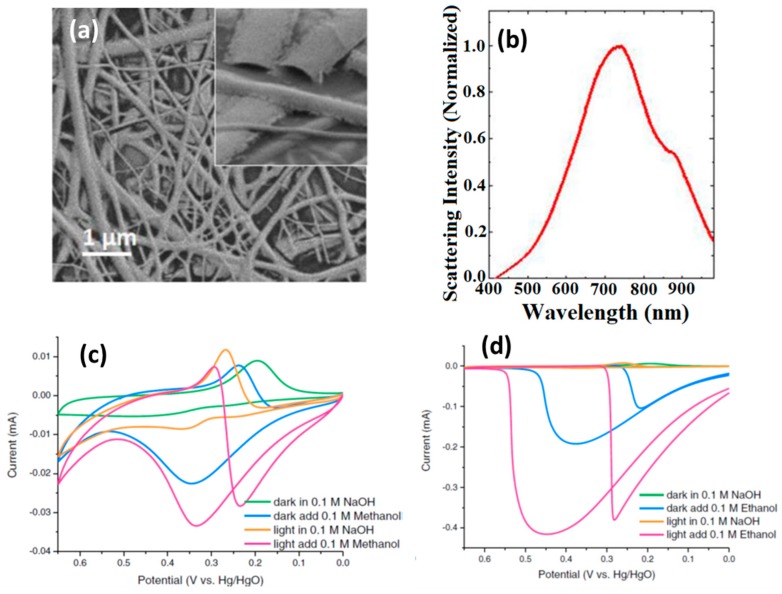
(**a**) SEM image of an Au nanofiber plasmonic electrode; (**b**) UV/Vis spectrum of Au nanofiber electrode; (**c**) cyclic voltammograms of Au nanofiber electrode under light or in the dark recorded in NaOH (0.1 M) in the presence or absence of methanol (0.1 M); (**d**) cyclic voltammograms of Au nanofiber electrode under light or in the dark recorded in NaOH (0.1 M) in the presence or absence of ethanol (0.1 M) (reprinted with the permission of Ref. [11]).

Silver is another promising material for plasmon-enhanced electrocatalysis due to its high extinction cross-section, but has not been reported so far, which is probably due to the low chemical stability of Ag. Hybrid bimetallic plasmonic nanostructures allow not only a high degree of control over the LSPR decay mechanism [28], but can help to overcome stability issues. Ag–Au nanoparticles were for example used for the plasmon-enhanced electrocatalytic oxidation of glycerol [12]. (Table 1). An Ag–Au catalyst that had been synthesized by a sacrificial support method was proposed recently by Minteer et al. for the enhanced electrocatalytic oxidation of glycerol [12]. The metals were chosen as they are well-known plasmonic materials with LSPR bands in the visible region of the electromagnetic spectrum, and are both able to electrocatalytically oxidize alcohols. When the binary Ag–Au catalyst—immobilized onto carbon-based anode—was illuminated with visible light, a significant increase in current and power output was observed (Figure 3), with an average current density under illumination of 280 µA cm^−2^ correlating to a power density of 15 µW cm^−2^. That no heating effect was observed under electrode illumination is in agreement with the concept of hot-electron transfer to glycerol.

Recently, surface plasmon-enhanced ethylene glycol electrooxidation was conducted by Xu et al. using hollow Pt–Ag nanodendrimers [29]. A 1.7-fold enhancement in catalytic activity under visible light irradiation compared to that under dark conditions was achieved. In addition, 6.2-fold and 7.0-fold enhancements when compared to commercial Pt/C were obtained when the optimized Pt–Ag nanostructures were employed as a photoelectrocatalyst. Indeed, while platinum (Pt) remains one of the most active catalysts for various electrochemical reactions, Pt NPs hardly exhibit an LSPR peak in the visible-light region, limiting their application for plasmon-induced electrochemical applications. The combination of Pt with Ag or Au NPs in the form of bimetallic nanoparticles has thus been investigated in several works [13,29,30,31].

One approach to increase the performance of a catalytic system is via the incorporation of special metal nanostructures, such as metal-tipped, porous, or needle-like plasmonic structures that have a high electrochemical surface area. Wei et al. recently proposed plasmonic bimetallic structures based on Pt-tipped Au nanorods (Au NRs) for electrochemical water splitting in the visible and the near-infrared region [32]. They demonstrated that these nanostructures outperformed fully Pt-covered nanostructures, which show weak LSPR bands. Pd–Ag hollow nanoflowers have been examined by Du et al. for the electrooxidation of ethylene glycol [33]. Pd atoms were deposited onto the surface of citrate-stabilized Ag seeds during a reducing agent-mediated galvanic replacement process with an electrochemical active surface area, which was determined as 25.8 m^2^ g^−1^ for a Pd_1_Ag_3_–hollow nanostructures, while Pt only exhibits 9.8 m^2^ g^−1^.

### 3.2. Metal–Semiconductor Composites

**Figure 4:**
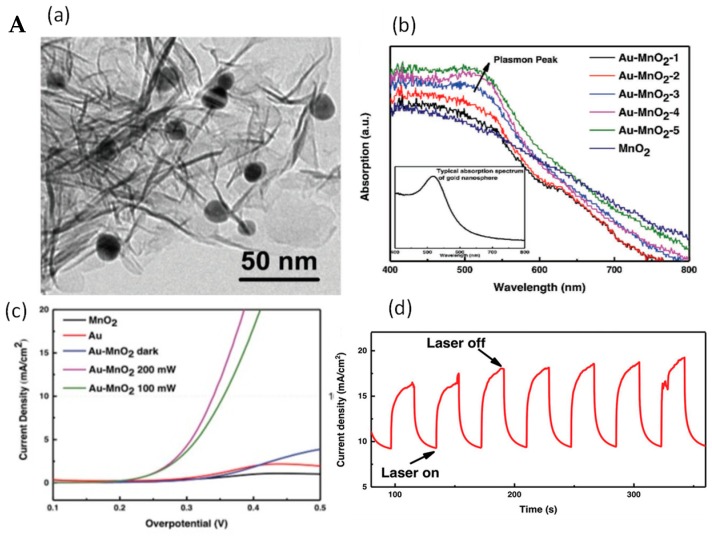

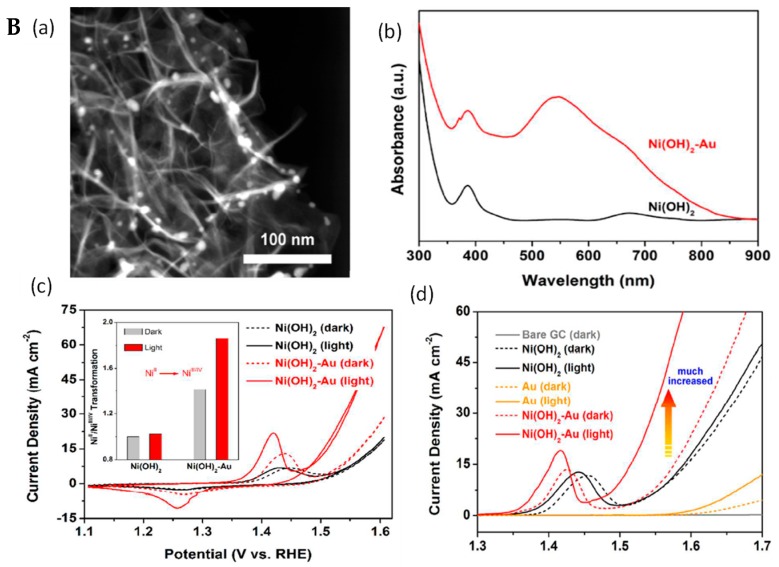
(**A**) (**a**) TEM image of Au–MnO_2_ nanocomposite, (**b**) UV/Vis absorption spectra of MnO_2_ nanosheets and Au–MnO_2_ nanocomposites with various Au loading (inset: LSPR band of gold nanospheres), (**c**) Polarization curve in 0.1 M of KOH with and without 532-nm laser irradiation, (**d**) Chronoamperometric I–t curve of Au@MnO_2_ nanocomposites with 532-nm laser on and off (reprinted with the permission of Ref. [9]); (**B**) (**a**) HAADF-STEM image of Ni(OH)_2_–Au hybrid catalyst; (**b**) UV/Vis absorption spectra of Ni(OH)_2_ nanosheets and Ni(OH)_2_–Au hybrid catalyst, (**c**) cyclic voltammograms with and without light irradiation of Ni(OH)_2_ nanosheets and Ni(OH)_2_–Au hybrid catalyst, and (**d**) oxygen evolution reaction (OER) polarization curves at 10 mV s^−1^ for different electrodes in dark and under light irradiation (532-nm laser, 1.2 W) in 1 M KOH; Ag/AgCl (Saturated KCl) was used as reference electrode (reprinted with the permission of Ref. [8]).

One of the most widely used semiconductors for photocatalytic applications is titanium, TiO_2_, which is low-cost, non-toxic, and has a stable wide band gap (3.2 eV) semiconductor, for which UV light is needed for practical applications. One way to overcome the large energy barrier of TiO_2_ is through its hybridization with noble plasmonic nanostructures absorbing in the visible region [34,35,36,37]. One of the first reports was proposed by Xu et al. [36], who decorated highly ordered TiO_2_ nanotube arrays (TiO_2_ NTs) with Au NPs (1.9 at.%) for enhanced ethanol oxidation. To further enhance the catalytic reaction, a bilayer titanium dioxide nanotube (BTNT) decorated periodically with Au NPs was proposed [35]. This heterostructured plasmonic electrode allowed ethanol electrooxidation under visible light illumination with a maximum catalytic current reaching 1.11 mA cm^−2^, which was 3.6-fold higher than that of conventional Au NPs-decorated monolayer TiO_2_ nanotubes. Placing Au NPs into TiO_2_ nanocavity arrays resulted in plasmonic electrochemical interfaces with superior oxygen reduction reaction (ORR) activity [34]. It was demonstrated that a 5 nm gold layer deposited on TiO_2_ delivered a superior ORR performance with an onset potential of 0.92 V versus reversible hydrogen electrode (RHE), a limiting current density of 5.2 mA cm^−2^, and an electron transfer number of 3.94. The enhanced reductive activity is attributed to the LSPR effect of isolated Au NPs in TiO_2_ nanocavities which suppressed electron recombination. MnO_2_ nanosheets decorated with Au NPs were reported by Xu et al. to be an ideal plasmonic electrocatalyst for the oxygen evolution reaction (OER) (Figure 4A) [38]. The confinement of the outer electrons of the Mn cations by plasmonic “hot holes” that were generated on the Au NPs’ surface was largely promoted under green light illumination. These hot holes act as efficient electron traps to form active Mn^n+^ species, providing active sites to extract electrons from OH^−^ and eventually facilitate OER catalysis.

Transition-metal catalysts [8] and metal–organic frameworks (MOFs) [10] are attractive alternatives for the oxygen evolution reaction. Liu et al. demonstrated that when Au NPs are decorated with transition-metal catalysts, such as Ni(OH)_2_ nanosheets (Figure 4B), they form a plasmonic electrocatalyst, which, upon light illumination, enhances the charge transfer from Ni(OH)_2_ to Au NPs, and greatly facilitates the oxidation of inactive Ni^2+^ to active Ni^3+/4+^ species, allowing more efficient water oxidation at a lower onset potential [8].

Transition-metal disulfides such as MoS_2_, on the other hand, are among the attractive alternatives for catalyzing hydrogen evolution reactions (HERs) [26]. To overcome the limitation of inherently low interparticle conductivity, Shi et al. proposed the incorporation of Au NRs in MoS_2_ [26]. The authors found that Au@MoS_2_ hybrids drastically improve the HER, with a three-fold increase of the current under excitation of the Au LSPR bands.

## 4. Current Trends and Outlook

This short review summarizes the recent developments on nanomaterials for plasmon-enhanced electrochemical reactions with the aim of interesting the communities working in plasmonic and electrochemical processes, providing a common base for jointly progressing in this exciting area of plasmon-mediated electrochemistry. To understand better the design of the nanostructures, the physical fundamental of localized surface plasmon resonance and the various mechanisms for plasmon-enhanced electrochemistry have been provided. Despite the significant advances achieved in the last three years, researchers are facing many challenges in this field. While a large variety of synthetic methods have been developed for the synthesis of these heterostructures, the scale-up of such processes will be an important and imperative aspect for the use of these concepts on a wider scale. The formation of highly reproducible nanostructures with comparable catalytic and plasmonic properties is at the core of the development to envision scale-up, which is an issue that has not been yet resolved. While laser light is often used for the stimulation of the plasmonic effect, developing plasmonic materials that are responsive to sunlight with high catalytic activity represents an important goal in the field of plasmon-mediated chemical/electrochemical reactions. Which guidelines for the future design of plasmonic electrochemical materials can be provided? The formations of bimetallic and metal/semiconductor nanostructures have both shown to be of great promise for plasmon-enhanced electrochemical systems, taking advantage of the catalytic activity and the strong optical effects of these nanostructures. While such simple plasmonic nanostructures have been demonstrated as concentrating light efficiently from the UV to the near-infrared range of the light spectrum and transferring it to adjacent species, thus improving electrochemical transformations, a better understanding of the underlying mechanism of plasmon-enhanced electrochemistry is primordial for optimizing electrochemical-based oxidation and reduction processes. How to separate the generated hot electrons from the holes in an efficient and controlled manner is one of the critical criteria to be investigated, as it is fundamental for enhancing electrochemical reactions. Next to this, the importance of plasmonic heating in plasmon-enhanced electrocatalysis has to be systematically studied. How to distinguish the contribution from the electromagnetic field-enhancement from hot charge carriers-induced enhancement are big experimental and theoretical challenges to be addressed. Further studies focusing on the morphology, composition, heterojunctions, and other nanomaterials-based aspects need to be conducted in order to fully optimize the plasmon-enhanced electrochemical effects. The future of plasmon-mediated electrochemistry might be full of surprises.

## Figures and Tables

**Table 1:** Plasmon-mediated electrochemical catalysis.

Plasmonic Catalyst	Electrode	Reaction	Electrolyte	Comments	Ref.
**Noble metal-based plasmonic electrocatalysts**					
Au NPs	GCE	glucose oxidation	PBS (pH 13.7)	High alkaline conditions to scavenge holes by OH^−^	[4]
Au nanofiber	GCE	ethanol and methanol oxidation	0.1 M NaOH	Decreased passivation effects	[11]
Ag–Au NPs	GCE	glycerol oxidation	0.1 M NaOH	100% fuel cell power output under visible light	[12]
Au NPs, Au NRs, Au NSs	GCE	ascorbic acid oxidation	PBS (pH 7.4)	Au NPs have weakest effect	[5]
Pt–Ag dendrites	GCE	ethylene glycol oxidation	1.0 M KOH	1.7-fold increase in catalytic activity under light	[29]
Au–Pt NPs	FTO	ethanol oxidation	1.0 M NaOH	2.6 times enhancement	[30]
Ag–Pt nanocages	GCE	ORR	0.1 M KOH	“Hot” electron transfer suppressed formation of peroxide intermediate	[13]
Pt/Fe–Au NRs	GCE	HER	0.5 M H_2_SO_4_ 1.0 M KOH	Photothermal effect results in electrocatalysis enhancement	[31]
Pd-tipped Au NRs	GCE	HER	0.5 M H_2_SO_4_	High exchange current density of 1.585 mA/cm^2^	[32]
PdAg hollow nanoflowers	GCE	Ethylene glycol oxidation	1.0 M KOH	High active surface area of 25.8 m^2^ g^−1^ (Pt 9.8 m^2^ g^−1^)	[33]
**Plasmonic metal–semiconductor composites**					
Au–TiO_2_	GCE	ORR	0.1 M NaOH	Activity of 310 mA mg^−1^	[34]
Au–TiO_2_ nanotubes	Ti foil	ethanol oxidation	0.5 M H_2_SO_4_ 1.0 M KOH	3.6-fold increase with low Au NPs (1.9 at.%)	[35]
Au–MnO_2_ NPs	GCE	OER	0.1 M KOH	60-mV overpotential	[9]
Ni(OH)_2_–Au	GCE	OER	1 M KOH	Four-fold enhancement, Tafel slope of 35 mV dec^−1^	[8]
Au–Co/NiMOF	GCE	OER	1 M KOH	10-fold increase	[10]
Au–CuI NPs	GCE	ethanol oxidation and methylene blue (MB) degradation	1 M KOH	5.6 (ethanol) and 13 times (MB) enhanced activity.	[39]
Au–MoS_2_	GCE	HER	0.5 M H_2_SO_4_	∼three-fold increase, turnover of 8.76 s^−1^ at 300 mV	[26]
TiN and doped graphene	GCE	HER	0.5 M H_2_SO_4_	Attained an HER current density of 10 mA/cm^2^ at a low overpotential of 161 mV.	[14]
Au NP@rGO layer@Pd NS	GCE	Water splitting (OER and HER)	0.1 M KOH	Under visible light irradiation 1.9 and 1.1-fold enhanced HER and OER activity, respectively.	[40]

Au NPs: gold nanoparticles; Au NRs: gold nanorods; Au NSs: gold nanostars, HER: Hydrogen evolution reaction; ORR: Oxygen reduction reaction; OER: Oxygen evolution reaction; GCE: glassy carbon electrode; FTO: Flourine doped tin oxide; MB: Methylene blue; MOF: metal-organic frameworks; PBS: phosphate-buffered saline.
